# Emergency department use and hospitalizations among homeless adults with substance dependence and mental disorders

**DOI:** 10.1186/s13722-015-0038-1

**Published:** 2015-08-05

**Authors:** Adrienne Cheung, Julian M Somers, Akm Moniruzzaman, Michelle Patterson, Charles J Frankish, Michael Krausz, Anita Palepu

**Affiliations:** Department of Medicine, Centre for Health Evaluation and Outcome Sciences, University of British Columbia, 588B-1081 Burrard Street, Vancouver, BC V6Z 1Y6 Canada; Faculty of Health Sciences, Simon Fraser University, Vancouver, Canada; School of Population and Public Health, Vancouver, Canada; Department of Psychiatry, University of British Columbia, Vancouver, Canada

**Keywords:** Emergency department use, Hospital admission, Substance dependence, Homelessness, At Home study, Mental disorders

## Abstract

**Background:**

Homelessness, substance use, and mental disorders each have been associated with higher rates of emergency department (ED) use and hospitalization. We sought to understand the correlation between ED use, hospital admission, and substance dependence among homeless individuals with concurrent mental illness who participated in a ‘Housing First’ (HF) intervention trial.

**Methods:**

The Vancouver At Home study consisted of two randomized controlled trials addressing homeless individuals with mental disorders who have “high” or “moderate” levels of need. Substance dependence was determined at baseline prior to randomization, using the Mini International Neuropsychiatric Interview diagnostic tool, version 6.0. To assess health service use, we reviewed the number of ED visits and the number of hospital admissions based on administrative data for six urban hospitals. Negative binomial regression modeling was used to test the independent association between substance dependence and health service use (ED use and hospitalization), adjusting for HF intervention, age, gender, ethnicity, education, duration of lifetime homelessness, mental disorders, chronic health conditions, and other variables that were selected a priori to be potentially associated with use of ED services and hospital admission.

**Results:**

Of the 497 homeless adults with mental disorders who were recruited, we included 381 participants in our analyses who had at least 1 year of follow-up and had a personal health number that could be linked to administrative health data. Of this group, 59% (n = 223) met criteria for substance dependence. We found no independent association between substance dependence and ED visits or hospital admissions [rate ratio (RR) = 0.85; 95% CI 0.62–1.17 and RR = 1.21; 95% CI 0.83–1.77, respectively]. The most responsible diagnoses (defined as the diagnosis that accounts for the length of stay) for hospital admissions were schizo-affective disorder, schizophrenia-related disorder, or bipolar affective disorder; collectively reported in 48% (n = 263) of admissions. Fifteen percent (n = 84) of hospital admissions listed substance dependence as the most responsible diagnosis.

**Conclusions:**

Substance dependence was not independently associated with ED use or hospital admission among homeless adults with mental disorders participating in an HF trial. Hospital admissions among this cohort were primarily associated with severe mental disorders.

Trial registration: ISRCTN57595077 and ISRCTN66721740

## Background

Homelessness is associated with a number of health and policy challenges in urban settings around the world. A significant proportion of individuals struggling with homelessness also suffer from substance dependence and other concurrent mental illnesses [1, 2]. A higher prevalence of these disorders has been observed among homeless populations in Canada, the United States, Europe, and Australia [3, 4]. In Toronto, Canada, lifetime diagnosis of mental illness and substance use or dependence has been recorded as high as 67 and 68%, respectively, among users of homeless shelters [1]. In the Hotel Study, which included 297 adults living in single-room occupancy hotels in Vancouver, the lifetime prevalence of mental illness and substance dependence was 98 and 85%, respectively [5].

Homelessness, substance use, and mental disorders each have been associated with higher rates of emergency department (ED) use, hospitalization, and involvement with other publicly-funded services in the United States and Canada [6–10]. One survey of 2,500 adults who were homeless in the United States found that less stable housing, history of psychiatric hospitalization, and substance abuse were associated with repeated use of ED services (defined as four or more visits in the previous year) [10]. Tsai et al. reported similar findings in comparing homeless to housed persons accessing Veterans Affairs EDs in the U.S. Homeless veterans had four times the odds of using the ED as housed veterans, and they were also more likely to have been diagnosed with a substance use disorder or schizophrenia in the preceding year [11]. Another study, examining discharge data from New York City hospitals, found that patients who were homeless stayed in the hospital, on average, 4.1 days (36%) longer than a comparison group [12]. Using the same sample as the present study, Palepu et al. found that daily substance use was associated with more severe mental health symptoms [2], which could lead to increased health care utilization. It follows that interventions addressing substance use and mental disorders among the homeless may reduce downstream health care expenditures.

Housing First (HF) is a low-barrier intervention designed to target the most vulnerable among the homeless, including those with severe mental illness experiencing chronic homelessness [7, 13]. HF provides immediate access to subsidized housing with supports and does not impose prerequisites of abstinence from substance use or adherence to medication for mental disorders. The model prioritizes consumer choice and an individual’s rights to appropriate housing, with the proposition that helping to meet immediate needs will enable the patients to address addiction and other psychiatric conditions. In addition to the housing component, different methods of support and treatment are offered to support recovery in a more effective way [13, 14]. These services have multiple goals, including the reduction of unnecessary hospitalizations and ED visits. We found that the assertive community treatment intervention of HF was associated with a reduction in ED visits [15].

Positive outcomes have been observed with HF in homeless adults with concurrent disorders, including greater residential stability and greater perception of choice among participants [16–18]. A number of studies have reported reductions in health service use and health care costs with HF interventions, including reductions in ED visits, hospitalizations, and length of hospital stay [7, 19–21]. Contrary to these findings, however, Hwang et al. found no difference in health service use between a group of supportive housing program participants and a control group in a study conducted in Toronto [21]. Another study, following a cohort of applicants to a supportive housing program in the United States, also failed to find reductions in health care use over time; this retrospective cohort study showed no significant difference in utilization rates of ambulance services and the ED before and after the intervention, and no difference was detected between the intervention group and a wait-list control group [22]. Although promising results have been published regarding the effectiveness of HF, some controversy remains as to its effect on participants’ use of health services.

In spite of the documented higher prevalence of substance use among the homeless, evidence is lacking on the effect of substance dependence on various outcomes, such as health service use for this group [23–25]. The objective of this study was to examine whether substance dependence at baseline predicted health service use at 2-year follow-up among participants assigned to HF or to treatment as usual (TAU), using data from the Vancouver At Home (VAH) study. We were also interested in whether or not substance dependence altered the effect of HF on ED use and hospitalization. We hypothesized that persons who were homeless and met criteria for substance dependence would have significantly higher levels of ED use and hospital admission at 2-year follow-up compared to persons without substance dependence.

## Methods

The detailed methods for the At Home/Chez Soi collaboration and the VAH study have been previously described [13, 14]. Essentially, At Home/Chez Soi is a pragmatic, multisite randomized controlled trial (RCT) assessing the effectiveness of a complex housing and support intervention in five Canadian cities. The VAH study includes additional measures, a site-specific intervention, and has a particular focus on substance use. In this manuscript, we report findings from the VAH study using survey data from participants recruited between October 2009 and June 2011, and administrative data on ED use and hospitalization spanning April 2007–September 2012. The VAH study is comprised of two RCTs examining the effectiveness of HF interventions among homeless adults with mental disorders who were differentiated based on their assessed levels of need (high vs. moderate). We pooled data from the two trials (ISRCTN57595077 and ISRCTN66721740) to examine the relationship between substance dependence and ED use and hospitalization [14]. Institutional Research Ethics Board approval was received from Simon Fraser University and the University of British Columbia.

Participants were recruited through referrals from a range of community agencies including shelters, drop-in centers, homeless outreach teams, mental health teams, inpatient hospital wards, and criminal justice programs [13, 14]. Individuals were eligible if they were age 19 years or older, met criteria for a current mental disorder on the Mini International Neuropsychiatric Interview (MINI) 6.0 [26], with or without concurrent substance dependence, and were either absolutely homeless or precariously housed.

Initial screening with referring service providers was conducted over the telephone [14]. This was followed by a face-to-face screening interview with the potential participant, where trained interviewers determined eligibility, explained study procedures, and obtained informed consent. A total of 800 individuals were screened for eligibility. Approximately 100 individuals did not meet eligibility criteria in the telephone screen. Another 200 were excluded through the baseline interview procedure due to ineligibility (n = 94), the inability to be contacted for baseline interview (n = 100), declining to participate (n = 3), or an incomplete interview (n = 3).

Once participants were enrolled, interviewers administered a baseline questionnaire, which consisted of detailed questions regarding sociodemographic characteristics, symptoms of mental disorders, substance use, physical health, and service involvement [14]. Participants received an honorarium of $35 upon completing the baseline interview. Participants were identified as high needs (HN) if they had a score of 62 or lower on the Multnomah Community Ability Scale [27], met criteria for current psychotic disorder or a (hypo)manic episode on the MINI, and had at least one of the following: two or more hospitalizations for mental illness in any one of the last 5 years, substance dependence in the past month, or legal involvement in the past year [13, 14]. All other included participants were designated as moderate needs (MN).

A detailed description of the intervention arms has been published previously [14]. Briefly, HN participants were randomized to one of three intervention arms: (1) HF with assertive community treatment (HF-ACT), where participants were given a choice of up to three market rentals and had to fulfill the commitments of their lease and check in with an ACT team member on a weekly basis with a client/staff ratio of 9:1; (2) HF in a congregate housing unit (HF-CONG) with onsite support; or (3) HF with treatment as usual (HN-TAU), which provided no additional housing or support aside from what was available in the community. MN participants were randomized to one of two intervention arms: (1) HF with intensive case management (HF-ICM), where participants were given a choice of up to three market rentals and connected to existing community services through case managers and a client/staff ratio of 16:1; or (2) MN-TAU.

Randomization was computer-generated at a national data center using an adaptive randomization procedure. This allowed for sequential allocation of participants immediately after enrollment, without affecting the predictability of future assignments. Blinding of participants was impossible, and blinding of interviewers was not feasible as interviewers were required to obtain data on participants’ housing status. Interviewers met with participants at 3-month intervals over the 2-year follow-up period. Participants were asked to provide consent to access administrative hospital data through their provincial personal health numbers.

### Variables of interest

Our primary outcome was health service use, defined as the number of ED visits and hospitalizations during the observation period. Both ED use and hospital admissions were captured through the administrative data. This administrative data included information related to ED visits (such as number and type of ED visits, mode of arrival, name of hospital, chief complaint, discharge diagnosis, and disposition) and hospitalizations (such as number of hospital admissions, name of hospital, most responsible diagnosis) from April 2007 to September 2012 at six urban hospitals in the Vancouver Coastal Health Authority.

Our primary independent variable, substance dependence (yes/no), was identified at baseline using the MINI 6.0. Self-reported frequency of substance use over the past month was captured using the Maudsley Addiction Profile at baseline and every 6 months thereafter up to 24 months [28]. Drug use frequency was dichotomized to compare daily substance use versus nondaily use (less than daily or none) [2]. HF intervention was the combination of the three housing intervention arms (HF-ACT, HF-CONG, HF-ICM), and TAU was comprised of the two TAU arms (HN and MN). Mental health symptoms and severity were collected through the Colorado Symptom Index (CSI) [29, 30]. Sociodemographic information (including gender, self-reported race/ethnicity, marital status, highest educational attainment, and monthly income) was collected at baseline.

For mental disorders, the severe cluster included at least one current psychotic disorder, mood disorder with psychotic features, and (hypo)manic episode, as identified through the MINI [26]. The less-severe cluster included at least one current major depressive episode, panic disorder, or post-traumatic stress disorder. Based on a list of 31 chronic health conditions, participants were also asked to report any conditions that were expected to last or already had lasted at least 6 months. Chronic health conditions listed in the survey tool were adapted from the Canadian Community Health Survey [31] and the National Population Health Survey [32]. Positivity for blood-borne infectious diseases was obtained by self-report. Participants were asked three questions pertaining to access to care that we included in our analyses: (1) do you have a regular medical doctor? (2) Is there a place you go when you are sick or need advice about your health? (If answered yes, named a hospital ED); (3) in the past 6 months, was there ever a time when you felt that you needed health care but you didn’t receive it?

### Statistical analysis

We pooled data from the two trials for the analyses. We used descriptive statistics to characterize the sample (mean and SD for continuous variables, and proportion for categorical variables). We compared variables between groups using parametric (Student’s *t*-test or one-way ANOVA for continuous variables) and nonparametric (Pearson’s Chi square test for categorical variables) tests, as appropriate. We estimated the rate of ED visits and hospitalizations by dividing the total number of occurrences (visits or admissions) by the total follow-up time (person-years). We fit separate models for the number of ED visits and the number of hospitalizations. We used negative binomial regression (NBR) analysis to estimate the association between each outcome variable and the primary independent variable (substance dependence). We chose NBR due to over-dispersion, the count nature of outcome data, and better goodness of fit statistics compared to Poisson regression. Post randomization period (exposure time) was estimated from the differences between time 0 (date of randomization) and time 1 (date of death or administrative data cutoff date, September, 2012), which varied across individuals (range 1.1–2 years). We used this exposure time (using the log transformation) as an offset variable in the regression analysis to adjust for these variations across individuals.

We examined the effects of substance dependence on health care use in both bivariate and multivariable settings. For the multivariable regression models, we included variables that were selected a priori to be potentially associated with ED visits and hospital admission [HF intervention (combined HF-ACT, HF-ICM, HF-CONG), need-level (HN vs. MN), employment, age, gender, ethnicity, education, age at first homelessness, mental disorders, chronic health conditions (3 or more), blood-borne infectious disease, prior health care utilization, having a regular doctor, and where one goes when sick]. In the model-building process, we chose all variables that were significant in bivariate models (p ≤ 0.05). In addition, we forced several demographic variables and substance dependence into the multivariable models, regardless of significance in bivariate models. We tested the interaction term between HF intervention and substance dependence, but did not include it in the final model due to nonsignificance (p > 0.05). We also conducted two sub-analyses fitting NBR models: one for the association of daily substance use and ED use and hospitalization using a similar set of covariates; and another model examining the association of substance dependence on psychiatric hospitalization. All reported p values were two-sided. Rate ratios (RRs) obtained from the NBR models were reported as effect sizes. The missing values for covariates that ranged from zero to 2% were excluded from the analysis. IBM SPSS statistics (version 19.0, August 2010) and STATA 12 (StataCorp. 2011) were used to conduct these analyses.

## Results

The eligible sample consisted of 381 participants who had at least 1 year of follow-up, provided consent to access administrative health data, and had a personal health number that could be used to access those data. There were no significant differences in the characteristics of the eligible sample compared to the total sample (Table 1). Among the eligible sample, 59% (n = 223) met criteria for substance dependence, and 29% (n = 110) reported daily substance use. Twenty-eight percent (n = 105) of the sample was female, and 16% (n = 62) identified as Aboriginal. Seventy percent (n = 266) of participants reported having at least three or more chronic health conditions, and 32% (n = 121) reported having a blood-borne infectious disease (HIV, hepatitis B, or hepatitis C). Most participants (67%) reported having a regular medical doctor. The average follow-up time was 1.94 years (SD 0.15 years, range 1.1–2 years). Tables 2 and 3 present the participant characteristics by ED and hospital admission rate (per person, per year).

**Table 1:** Characteristics of “At Home” participants by Housing First allocation status

Variable	Entire sample (N = 497) n (%)/mean (SD)	Eligible sample (n = 381) n (%)/mean (SD)	Housing First-yes (n = 250) n (%)/mean (SD)	Housing First-no (n = 131) n (%)/mean (SD)	p value^a^
Housing First interventions	297 (60)	250 (66)			
Substance dependence	288 (58)	223 (59)	150 (60)	73 (56)	0.421
Daily substance use	143 (29)	110 (29)	78 (31)	32 (25)	0.172
Need level (high)	297 (59)	223 (59)			
Age at randomization (in years)	40.8 (11.0)	40.6 (10.9)	40.6 (10.8)	40.6 (11.1)	0.968
Age of first homelessness (in years)	30.3 (13.3)	29.9 (13.4)	29.5 (12.9)	30.5 (14.2)	0.477
Female gender	134 (27)	105 (28)	65 (26)	40 (31)	0.313
Aboriginals	77 (16)	62 (16)	45 (18)	17 (13)	0.119
White	280 (56)	206 (54)	139 (56)	67 (51)	
Other	140 (28)	113 (30)	66 (26)	47 (36)	
Education (incomplete high school)	280 (57)	214 (57)	147 (60)	67 (51)	0.118
Single/never married	343 (70)	254 (67)	168 (68)	86 (66)	0.755
Income ($800 CDN or more)^b^ in past month	257 (52)	201 (54)	138 (56)	63 (49)	0.180
Lifetime duration of homelessness (in months)	60.2 (70.3)	56.8 (62.2)	60.6 (66.4)	49.5 (52.7)	0.099
Longest episode of homelessness (in months)	30.9 (40.1)	29.7 (38.8)	31.1 (40.2)	27.2 (36.0)	0.353
Less severe cluster of mental disorders	264 (53)	201 (59)	130 (52)	71 (54)	0.683
Severe cluster of mental disorders	363 (73)	272 (71)	180 (72)	92 (70)	0.716
Multiple mental disorders (≥3)	114 (25)	88 (23)	61 (24)	27 (21)	0.405
Suicidality (high)	87 (17)	68 (18)	44 (18)	24 (18)	0.861
Mental health severity/CSI score (per unit)	37.2 (12.5)	37.3 (12.6)	36.6 (12.8)	38.7 (12.2)	0.116
Chronic health conditions (≥3)	344 (69)	266 (70)	172 (69)	94 (72)	0.551
Blood-borne infectious disease (HIV, hepatitis B or C)	157 (32)	121 (32)	83 (33)	38 (29)	0.416
Have a regular medical doctor	320 (65)	257 (67)	170 (68)	87 (67)	0.831
Place to go when you are sick	395 (81)	302 (81)	202 (82)	100 (78)	0.396
Needed health care, but didn’t receive it	209 (43)	156 (42)	97 (40)	59 (46)	0.239
ED visit before randomization (last year)		4.1 (7.0)	4.2 (7.1)	4.0 (6.8)	0.778
Hospital admissions before randomization (last year)		0.9 (1.5)	0.9 (1.5)	0.9 (1.5)	0.805

^a^P values based on comparisons of characteristics between HF-yes participants and HF-no participants in the eligible sample (n = 381).

^b^Dichotomized based on median value.

**Table 2:** ED visit during the post randomization period, by “At Home” participant characteristics (n = 381)

Variable	# of ER visits	Total person-years (PYs)	ED rate (per person, per year)
Overall	3,086	738.2	4.2
Housing First interventions	1,925	482.8	4.0
Treatment as usual	1,161	255.4	4.6
Substance dependence			
Yes	1,784	430.9	4.1
No	1,302	307.3	4.2
Daily substance use			
Yes	921	213.3	4.3
No	2,165	524.9	4.1
Need level			
High	2,038	430.6	4.7
Moderate	1,048	307.6	3.4
Male	2,127	529.5	4.0
Female	947	202.7	4.7
Aboriginals	672	119.7	5.6
White	1,794	398.5	4.5
Other	620	219.0	2.8
Incomplete high school	2,008	416.5	4.8
High school or higher	1,062	315.7	3.4
Single/never married	2,225	493.2	4.5
Other	796	239.0	3.3
Income ($800 CDN or more) in past month			
Yes	1,660	387.0	4.3
No	1,413	339.5	4.2
Less severe cluster of mental disorders			
Yes	1,605	390.1	4.1
No	1,481	348.1	4.3
Severe cluster of mental disorders			
Yes	2,369	527.5	4.5
No	717	210.7	3.4
Multiple mental disorders (≥3)			
Yes	826	171.3	4.8
No	2,260	566.9	4.0
Suicidality (high)			
Yes	592	132.5	4.5
No	2,494	605.7	4.1
Chronic health conditions (≥3)			
Yes	2,426	516.6	4.7
No	660	221.6	3.0
Blood-borne infectious disease (HIV, hepatitis B or C)			
Yes	941	235.6	4.0
No	2,143	498.6	4.3
Have a regular medical doctor			
Yes	2,204	497.6	4.4
No	882	240.6	3.7
Place to go when you are sick			
Yes	2,757	595.2	4.6
No	328	143.0	2.3
Needed health care, but didn’t receive it			
Yes	1,568	321.3	4.9
No	1,518	416.9	3.6

**Table 3:** Acute hospital admissions during the post randomization period among “At Home” participants (n = 381)

Variable	Hospital admissions (N)	Total person-years (PYs)	Hospitalization rate (per person, per year)
Overall	550	721.9	0.8
Housing First interventions	367	471.6	0.8
Treatment as usual	183	250.3	0.7
Substance dependence			
Yes	338	421.2	0.8
No	212	300.7	0.7
Daily substance use			
Yes	124	207.9	0.6
No	426	514.0	0.8
Need level			
High	366	420.2	0.9
Moderate	184	301.7	0.6
Male	390	518.8	0.8
Female	157	197.1	0.8
Aboriginals	79	116.2	0.7
White	299	389.9	0.8
Other	172	215.8	0.8
Incomplete high school	355	407.1	0.9
High school or higher	195	308.8	0.6
Single/never married	410	482.0	0.9
Other	138	234.0	0.6
Income ($800 CDN or more) in past month			
Yes	319	377.9	0.8
No	229	332.6	0.7
Less severe cluster of mental disorders			
Yes	227	381.0	0.6
No	323	340.9	1.0
Severe cluster of mental disorders			
Yes	465	516.0	0.9
No	85	205.9	0.4
Multiple mental disorders (≥3)			
Yes	118	167.8	0.7
No	432	554.1	0.8
Suicidality (high)			
Yes	106	129.1	0.8
No	444	592.8	0.8
Chronic health conditions (≥3)			
Yes	352	506.6	0.7
No	198	215.3	0.9
Blood-borne infectious disease (HIV, hepatitis B or C)			
Yes	167	231.8	0.7
No	383	486.2	0.8
Have a regular medical doctor			
Yes	324	484.0	0.7
No	226	236.0	1.0
Place to go when you are sick			
Yes	460	570.8	0.8
No	88	140.7	0.6
Needed health care, but didn’t receive it			
Yes	198	299.5	0.7
No	335	406.1	0.8

The 381 participants incurred a total of 3,086 ED visits during the 2-year study period. The average number of ED visits was 4.2 per person, per year (Table 2). Less than one-quarter (23%) had no visits and one individual accumulated 176 ED visits. The multivariable NBR model (Table 4) showed no significant association between substance dependence and ED visits [adjusted incidence rate ratio (ARR) = 0.85; 95% CI 0.62–1.17]. The HF intervention was associated with a reduction in subsequent ED visits (ARR = 0.74; 95% CI 0.55–1.00). We found that having an ED visit in the year prior to randomization (ARR = 1.11; 95% CI 1.08–1.14) and reporting the ED as a place to go when sick (ARR = 1.45; 95% CI 1.01–2.09) were associated with higher rates of ED use. There were no significant interactions between substance dependence and the HF intervention on ED visits (p = 0.50) or between substance dependence and ACT on ED visits (p = 0.45).

**Table 4:** Negative binomial regression analysis to estimate the effect of substance dependence on ED visits during the post randomization period among “At Home” participants (n = 381)

Variable	Unadjusted RR (95% CI)	p value	Adjusted RR (95% CI)
Substance dependence (yes vs. no)	0.99 (0.73, 1.34)	0.953	0.85 (0.62, 1.17)
Housing First interventions (yes vs. no)	0.89 (0.65, 1.22)	0.468	0.74 (0.55, 1.00)
Need level (high vs. moderate)	1.37 (1.02, 1.86)	0.039	1.11 (0.79, 1.56)
Age at randomization (per year)	0.99 (0.98, 1.01)	0.265	1.00 (0.98, 1.02)
Age of first homelessness	1.00 (0.99, 1.01)	0.900	0.99 (0.97, 1.00)
Female gender	1.13 (0.81, 1.58)	0.459	1.03 (0.75, 1.70)
Aboriginals	1.94 (1.23, 3.05)	0.004	1.32 (0.95, 1.84)
White	1.60 (1.14, 2.24)	0.006	1.05 (0.65, 1.76)
Other	Reference		
Education (incomplete high school)	0.71 (0.53, 0.96)	0.027	0.78 (0.58, 1.05)
Single/never married	1.33 (0.97, 1.83)	0.080	
Income ($800 CDN or more) in past month	1.05 (0.78, 1.41)	0.762	
ER visit before randomization (last year)	1.12 (1.09, 1.15)	<0.001	1.11 (1.08, 1.14)
Less severe cluster of mental disorders	0.97 (0.72, 1.31)	0.861	0.98 (0.73, 1.32)
Severe cluster of mental disorders	1.28 (0.92, 1.78)	0.141	1.32 (0.91, 1.92)
Chronic health conditions (≥3)	1.56 (1.12, 2.16)	0.007	1.13 (0.79, 1.63)
Blood-borne infectious disease (HIV, hepatitis B or C)	0.93 (0.68, 1.29)	0.678	1.28 (0.92, 1.79)
Have a regular medical doctor	1.20 (0.87, 1.65)	0.264	1.06 (0.77, 1.46)
Place to go when you are sick	2.06 (1.41, 3.00)	<0.001	1.45 (1.01, 2.09)
Needed health care, but didn’t receive it	1.36 (1.01, 1.84)	0.046	0.93 (0.69, 1.27)

Participants in the eligible sample incurred a total of 550 hospital admissions during the 2-year follow-up period. The average number of admissions was 0.8 per person, per year (Table 3). The maximum number of admissions per person was 15. Sixty-one percent (n = 336) of admissions occurred during the first year post randomization. Psychiatric hospital admissions comprised 81% of the total hospitalizations (443/550) and were incurred by 137 homeless persons. The most responsible diagnosis for hospital admission was schizoaffective or schizophrenia-related disorder in 233 admissions (42%), followed by bipolar affective disorder in 30 admissions (6%). These diagnoses collectively accounted for 48% (n = 263) of hospital admissions, while 15% (n = 84) of admissions were attributable to substance use. As shown in Table 5, substance dependence was not independently associated with hospital admissions (ARR = 1.21; 95% CI 0.83–1.77). Higher rates of hospital admission were associated with having a hospital admission in the year prior to randomization (ARR = 1.33; 95% CI 1.19–1.49) and having a mental disorder in the severe cluster (ARR = 1.76; 95% CI 1.09–2.86). There was no significant interaction between substance dependence and the HF intervention on hospital admissions (p = 0.60).

**Table 5:** Negative binomial regression analysis to estimate the effect of substance dependence on acute hospital admissions during the post randomization period among “At Home” participants (n = 381)

Variable	Unadjusted RR (95% CI)	p value	Adjusted RR (95% CI)
Substance dependence (yes vs. no)	1.15 (0.81, 1.65)	0.433	1.21 (0.83, 1.77)
Housing first interventions (yes vs. no)	1.08 (0.75, 1.56)	0.682	0.65 (0.25, 1.72)
Need level (high vs. moderate)	1.42 (0.99, 2.03)	0.056	0.88 (0.58, 1.35)
Age at randomization (per year)	0.98 (0.97, 1.00)	0.044	0.99 (0.97, 1.01)
Age of first homelessness	1.00 (0.98, 1.01)	0.475	1.00 (0.98, 1.02)
Female gender	1.04 (0.70, 1.54)	0.844	1.05 (0.71, 1.55)
Aboriginals	0.83 (0.48, 1.44)	0.508	0.89 (0.51, 1.53)
White	0.97 (0.65, 1.45)	0.897	1.06 (0.73, 1.56)
Other	Reference		Reference
Education (incomplete high school)	1.35 (0.95, 1.93)	0.095	1.27 (0.88, 1.83)
Single/never married	1.42 (0.97, 2.08)	0.070	
Income ($800 CDN or more) in past month	1.23 (0.87, 1.75)	0.247	
Hospital admissions before randomization (last year)	1.34 (1.20, 1.50)	<0.001	1.33 (1.19, 1.49)
Less severe cluster of mental disorders	0.65 (0.46, 0.92)	0.015	0.76 (0.53, 1.10)
Severe cluster of mental disorders	2.12 (1.42, 3.17)	<0.001	1.76 (1.09, 2.86)
Chronic health conditions (≥3)	0.75 (0.51, 1.09)	0.127	0.99 (0.67, 1.46)
Blood-borne infectious disease (HIV, hepatitis B or C)	0.92 (0.63, 1.35)	0.682	
Have a regular medical doctor	0.70 (0.48, 1.01)	0.053	0.78 (0.54, 1.12)
Place to go when you are sick	1.28 (0.81, 2.01)	0.284	
Needed health care, but didn’t receive it	0.81 (0.57, 1.16)	0.260	

We did not find an association between daily substance use and health service use in the sub-analysis, when we fit separate models for the number of ED visits and the number of hospitalizations (data not shown). We also did not find a significant association between substance dependence and psychiatric hospitalization (ARR 1.14; 95% CI 0.73–1.80).

## Discussion

We found no association between substance dependence and health service use in the form of ED visits and hospitalizations. Daily substance use also was not associated with ED use or hospital admission in these models. Two studies also found no association between substance use and health service involvement [9, 33]. The first examined 2,974 homeless persons in the United States and did not find an association between alcohol and drug abuse with ED use or hospitalization [9]. The second study in Toronto also found no association between having an alcohol and drug problem with frequent ED visits (4.7 visits/year) [33]. In contrast, many studies have shown that homelessness, substance use, and mental disorders are all independently associated with higher rates of ED use and hospitalization [6–10, 22, 34, 35]. HF has been linked to increased residential stability and reductions in these health services in a number of studies [7, 19–21, 24, 25, 36], while others have found no significant association [21, 22]. Differences in these findings may be due to differences in the homeless samples in terms of the higher burden of medical and psychiatric comorbidities, severity of substance use, and health care systems in different jurisdictions. We found a reduction in ED visits among HF participants compared to the TAU group but did not detect a difference in hospitalizations, which is consistent with a finding previously reported among VAH high-needs participants in the HF-ACT arm that has been discussed elsewhere [15].

It is notable that our observed average rate of ED use was high, at 4.2 visits per year. This is in contrast to a recent study of 1,189 homeless adults who were followed for 4 years in Toronto [33]. Those researchers measured average ED visits per year at ~2, and they defined high utilizers as 4.7 visits per year, which corresponded to the top 10 percentile. Interestingly, the average ED visits among these frequent ED users was 12.1 per person-year. Unlike other studies of homeless persons, our criteria for study inclusion stipulated that they had to have a mental disorder, and 71% were classified as having severe cluster of mental disorders, including psychosis, mood disorder with psychotic features, and hypomanic or manic episode. Furthermore, 70% of our sample had at least three or more chronic health conditions, which is similar to a study of frequent ED use (>4 visits per year) among homeless veterans within the Veterans Affair health care system that found an association between the high burden of chronic medical and psychiatric diagnoses and frequent ED use [37]. Many other studies report lower annual rates of ED use among persons who are homeless, and those who had ≥4 ED visits annually are defined as frequent users [22, 38, 39]. In contrast, D’Amore et al. recorded an average ED visit rate of 6.2 per homeless person per year at one ED in New York City. This sample was similar to ours, with a high prevalence of mental health disorders and substance use [40].

In this study, we found no significant interaction between substance dependence and HF vs. TAU intervention on ED visits and hospitalization. Few studies have examined the effect of HF interventions in homeless populations with substance dependence [23]. It is reasonable to suspect that outcomes of HF may differ among this subgroup from the general homeless population, and further investigation is warranted considering the high prevalence of addiction among chronically homeless and mentally ill adults [16, 41]. Edens et al. examined health service costs in a population of active substance users, and Larimer et al. analyzed overall costs (including jail, ED, inpatient and outpatient contacts, emergency medical service calls, and transports) in chronically homeless adults with severe alcohol addiction. Both studies involved a low-barrier housing intervention similar to HF, and both studies found a reduction in costs when participants were stably housed [24, 25]. Martinez examined placement in permanent supportive housing and found that stable housing significantly reduced the percentage of residents with an ED visit, the average number of ED visits per person, and the total number of ED visits among homeless adults with substance use and mental disorders [36]. Our results support this existing research showing that HF can be equally effective in persons with and without substance dependence in reducing ED visits.

Of note, hospital admissions in this study were associated with the severe cluster of mental disorders, which accounted for 48% of hospital admissions in the follow-up period. Contrary to general perception, disorders attributed to substance use accounted for a relatively small proportion of hospital admissions (15%). Substance dependence also was not a driver for ED visits. In a study of New York City hospital discharge data, Salit et al. reported that 80.6% of hospital admissions among homeless adults involved either a principal or secondary diagnosis of substance use or mental illness, but did not report what proportion of admissions were attributed to substance use and mental illness independently [12]. It also may be that these results reflect the high level of stigma towards substance users among health professionals [42].

Hospitalization among homeless adults in Toronto was also recently examined [43], and 921 hospitalizations were incurred during the 4-year follow-up, of which 548 (59.5%) were medical or surgical and 373 (40.5%) were psychiatric. We observed 550 hospital admissions during the 2-year follow-up period and found a higher average yearly hospital admission rate (medical, surgical, and psychiatric combined) of 0.72 vs. 0.26 in their study. This is likely attributable to differences in the sample characteristics, where VAH had many more persons with severe mental disorders and did not include homeless families with children (who tend not to use as much health services). The Toronto researchers also found that a large proportion of the psychiatric admissions were for schizophrenia and other psychotic disorders and noted that some hospitalizations may be difficult to avoid [43].

These findings may be relevant to policymakers wishing to reduce health care expenditures among this population. Increasing availability and access to mental health services may reduce costly acute health service use more so than targeting substance dependence alone. One tertiary intervention that included a residential treatment program for persons with severe substance dependence and concurrent mental illness (of whom half were homeless at intake) found a reduction in substance use and psychopathology symptoms among those who completed the follow-up assessment at 6 months [44].

Several limitations should be considered when interpreting our results. The HF-CONG location was a few blocks away from a hospital, which may have influenced the frequency of ED use. It should be noted that given the high burden of mental disorders and chronic health conditions in this population, the provision of regular care may have identified the need for ED and hospital admissions, and the use of such services may have been appropriate. We would expect the use of these health services to decline with longer time in HF. We did not examine the effect of substance dependence within each HF intervention arm on ED visits and hospitalizations, given that the interaction term of substance dependence by HF was nonsignificant. Participants may also have been inclined to under-report substance use due to stigma and/or the fear of losing their housing/services. Access to administrative data provided a more accurate portrayal of health service use than self-reported measures; however, our analyses were based on incomplete data from the VAH study sample because some participants did not provide consent to access data, could not be linked to the database, or had less than 1 year of follow-up. Further, we were able to acquire data on ED visits and hospital admissions from six urban hospitals, but may have missed visits to other hospitals in British Columbia or outside of the province. Finally, the effect sizes observed in our multivariable analyses were generally small.

This study contributes to the body of evidence examining HF interventions in a homeless population with high rates of substance use and mental disorders, an area in which research is lacking. We analyzed data collected from an RCT as a longitudinal cohort with controls. This is an improvement on much of the previous research that has been based on cross-sectional or observational designs without controls. We were also able to achieve exceptionally high rates of follow-up among our participants.

## Conclusions

We found no significant association between substance dependence and health service use in the form of ED visits and hospital admissions. Hospital admissions in the VAH cohort were mainly associated with severe mental disorder diagnoses rather than substance use disorders, suggesting that exploring interventions to better optimize management of these categories of mental disorders may be key to reducing hospitalization, but may prove to be challenging among persons who are homeless with concurrent disorders. It is likely that any such intervention will need to be comprehensive and integrate both housing and social support in the long term.

## Appendix

See Table 6.

**Table 6:** Correlation between baseline substance dependence and daily substance use at each follow-up visit in the eligible sample (n = 381)

	All N (%)	SD-No N (%)	SD-Yes N (%)	p value
Daily substance use at baseline (n = 379)^a^	110 (29)	27 (17)	83 (37)	<0.001
Daily substance use at 6-month visit (n = 346)	93 (27)	24 (17)	69 (34)	<0.001
Daily substance use at 12-month visit (n = 357)	106 (30)	23 (16)	83 (39)	<0.001
Daily substance use at 18-month visit (n = 341)	89 (26)	24 (17)	65 (32)	0.003
Daily substance use at 24-month visit (n = 319)	75 (23)	21 (16)	54 (28)	0.014

^a^Participants with valid response to the questions related to daily substance use at each follow-up visit are presented in the parentheses. The remaining participants had either declined response or did not complete the interview.

## Abbreviations

HF: Housing First
ED: emergency department
VAH: Vancouver At Home
ACT: assertive community treatment
ICM: intensive case management
CONG: congregate
AU: treatment as usual
